# A robust model for simultaneously inducing corneal neovascularization and retinal gliosis in the mouse eye

**Published:** 2011-07-14

**Authors:** Riya R. Paranthan, Paola Bargagna-Mohan, Daniel L. Lau, Royce Mohan

**Affiliations:** 1 Ophthalmology & Visual Science, University of Kentucky, Lexington, KY; 2 Neuroscience, University of Connecticut Health Center, Farmington, CT; 3 Electrical & Computer Engineering, University of Kentucky, Lexington, KY

## Abstract

**Purpose:**

To develop an animal model for simultaneously eliciting corneal angiogenesis and retinal gliosis that will enable the assessment of inhibitor efficacy on these two pathological processes in separate anatomic sites of the ocular globe.

**Methods:**

Four to six week-old mice in a C57BL/6J background were anesthetized and 0.15 N NaOH was applied to the cornea, followed by mechanical scraping of the epithelium from limbus and central cornea. After this injury, mice were treated with vehicle or with an inhibitor (withaferin A [WFA]), which were delivered by intraperitoneal injection, to assess the pharmacological effects on angiogenesis and/or gliosis. Mice were sacrificed after 14 days and tissues (corneas and retinas) were prepared for analysis of corneal neovascularization and retinal gliosis by immunohistochemistry and western blotting, respectively. This protocol was also suited for studying earlier disease end points, for assessment of drug dose efficacy or genetic influences and the entire procedure and this analysis was completed in 16–17 days.

**Results:**

Both corneal angiogenesis and retinal gliosis were maximally sustained at fourteen days following chemical and mechanical injury of the cornea. 1) Injured corneas showed abundant CD31^+^ staining, with new blood vessels branching out from the limbus to the central cornea. WFA treatment potently inhibited corneal neovascularization. 2) Retinal gliosis in injured mice was associated with upregulated expression of glial fibrillary acidic protein (GFAP) that appeared as polymeric filaments and soluble forms expressed in reactive Müller glial cells. WFA treatment potently downregulated the expression of soluble and filamentous GFAP; the latter protein was fragmented.

**Conclusions:**

We have developed a mouse model for investigating retinal gliosis and corneal neovascularization. We used this model to demonstrate the simultaneous inhibitory effects of WFA on both of these disease processes. Retinal gliosis occurs in several major degenerative conditions of the eye, including age-related macular degeneration, where angiogenesis is also a prevailing pathological feature. Thus, inhibitors of both gliosis and angiogensis used as combination therapy are currently being explored for treatment of such complex diseases. The model presented here affords a very simple preclinical assay for screening combination of drugs or polypharmacological agents and reduces the numbers of animals because of the different anatomic sites of these pathologies. Finally, given that endogenous mediators elicit angiogenesis and gliosis in this model, the combination of genetics and pharmacology can be exploited to study drug mechanisms and for target validation in vivo.

## Introduction

Angiogenesis, the growth of new blood vessel from pre-existing vasculature [1,2] is an inherently beneficial process that occurs in physiologic processes, and gives tissues the ability to regenerate after injury or insult [3,4]. However, the imbalance of angiogenesis – either excess or insufficiency – is the hallmark of a vast number of diseases that target different organs [5]. For instance, the augmentation of angiogenesis contributes to cancer growth [6] and gives rise to chaotic vasculature in highly specialized organs such as the brain and eye [7,8].

Reactive gliosis is the proliferation and hypertrophy of astrocytes and glia in the central nervous system after injury [9,10]. A central hallmark of gliosis is the abundant increase in expression of type III intermediate filaments (IFs), protein glial fibrillary acidic protein (GFAP), and vimentin, which protect neurons from insult [11]. During gliosis, astrocytes accumulate into dense, fibrous patches called glial scars, which interfere with normal functioning of the central nervous system (CNS) [12], and which cause blindness when these scars affect the retina [13]. Gliosis is central to major retinal diseases such as age-related macular degeneration [14], diabetic retinopathy [15], and glaucoma [9] and is prevalent in many non-ocular diseases, including multiple sclerosis [16] and Alzheimer disease [17].

The eye is an excellent experimental model for the study of both angioproliferative diseases and gliosis because it comprises tissues that are highly specialized, yet still compartmentalized to retain their own physiologic identity. For instance, the cornea offers two extremely rare commodities: 1) transparency and 2) avascularity. For these reasons, it has been extensively used as a model for inducing neovascularization and for investigation of the efficacy of angiogenesis inhibitors [18]. In contrast, the retina is the best-understood sensory system of the vertebrate CNS [19]. In contrast to brain and spinal cord models of the development of traumatic injury in the CNS, where systemic morbidity and mortality occur, injury to the retina has localized effects and these are not lethal. In addition, the fellow eye of the model animal (typically a mouse) can serve as an internal control for testing effects of a drug on normal physiology or for evaluating toxic effects.

We have identified a class of type III IF inhibitor that shows polypharmacological activity on angiogenesis [20] and gliosis [21]. Withaferin A (WFA) is a small molecule natural compound that binds to vimentin and downregulates its expression, resulting in a blockade of corneal neovascularization through inhibition of endothelial cell proliferation, migration, and sprouting [22,23]. The WFA binding site is highly conserved in GFAP; consequently, WFA also potently inhibits astrocyte proliferation and attenuates retinal gliosis [21]. Both corneal angiogenesis and retinal gliosis can be robustly elicited by alkali injury with corneal scraping, which makes this a useful model for the study of angiogenesis inhibitors and anti-gliosis drug leads. In the present paper, we describe the use of this simple model and emphasize its use in drug assessment. A further benefit is that the trauma-initiating agent is alkali, which means that mice of any genetic background, as well as genetic knockout mice, can be used. This makes this model a convenient system for combining genetics and pharmacology for the simultaneous study of inhibition of angiogenesis and gliosis.

## Methods

### Materials

WFA was purchased from ChromaDex (Irvine, CA), solubilized in dimethyl sulfoxide (DMSO), and aliquoted in stock solutions that were stored at −20 °C. Vials were thawed immediately before use.

### Animals

Mice in C57BL/6J background were purchased from Jackson Laboratories (Bar Harbor, MA) and housed in pathogen-free cages at the Animal Core Facility of the University of Kentucky with a 12 h:12 h light-dark cycle. All animal experiments followed the guidelines of the Association for Research in Vision and Ophthalmology (ARVO) Statement for the Use of Animals in Ophthalmic and Vision Research, and procedures approved by Institutional Animal Care and Use Committee (IACUC) committee of the University of Kentucky. Lexington, KY.

### Alkali Burn Model of Corneal Angiogenesis and Gliosis

Mice were anesthetized and 1 μl drop of dilute 0.15 M sodium hydroxide was applied for 1 min to the central corneas of each mouse and immediately washed out with abundant saline solution. The corneal and limbal epithelium was gently removed by scraping with a blunt Tooke corneal knife and the cornea was topically treated with atropine eye drops. WFA (2 mg/kg solubilized in dimethyl sulfoxide [DMSO]) or vehicle (DMSO) was provided by intraperitoneal injection in respective drug or control groups of mice immediately after their recovery from anesthesia as done previously [20,21], and subsequently every day thereafter for 14 days. Mice were sacrificed by carbon dioxide asphyxiation and eyes enucleated immediately.

### Immunostaining

#### Retina staining

Whole eyes were embedded for frozen sectioning, and tissue sections (10 μm thick) were fixed for 5 min in ice-cold methanol and then permeabilized and blocked simultaneously in PBS buffer (PBS-T) containing 0.2% Triton-X, 1% BSA, and 5% goat serum at 37 °C for 1 h. Samples were then washed 3 times with PBS and incubated with anti-rabbit GFAP antibody (1:1000; Abcam, Cambridge, MA) for 18 h at 4 °C, as previously described [21]. After appropriate secondary antibody incubation, digital images of immunostained sections were acquired on a Nikon TE2000 (Nikon Instruments Inc., Melville, NY) microscope at 30× magnification (bars 50 μm).

#### Whole-mount cornea staining

Freshly isolated corneal buttons were carefully sectioned from the globe of injured and WFA-treated mice. Corneal buttons were fixed in 100% acetone for 20 min, washed in PBS for 1 h, and blocked for 3 days in 1% BSA-PBS at 4 °C. Corneas were incubated with FITC-conjugated anti-mouse CD31 antibody (1:300; BD PharMingen, San Diego, CA) at 4 °C for 12 h, washed and affixed to glass slides with a coverslip. Fluorescent staining was visualized with a Nikon TE2000 microscope, and quantified by importing digital images to NIH ImageJ [20].

#### Flatmount retina staining

Enucleated eyes were fixed in 4% paraformaldehyde (PFA) in PBS for 30 min at room temperature. After removal of corneas and lenses, the posterior part of the eyecups containing the choroid and retina were incubated in 4% PFA for another 30 min and tissue processed according to published procedures [22]. After careful washing, retinas were peeled off and permeabilized and blocked overnight in a PBS solution containing 0.5% Triton-X100 and 5% goat serum at 4 °C. After washing in PBS, retinas were incubated overnight with anti-rabbit GFAP (1:100 dilution in 0.5% Triton-X100, and 5% goat serum solution) at 4 °C. After washing in PBS for 30 min, retinas were incubated for 1 h at room temperature with secondary antibody conjugated to AlexaFlour-488 (1:1000 dilution in 0.5% Triton-X100, and 5% goat serum solution). Retinas were mounted onto slides with the vitreous side facing upwards. Images used to compile the montage panel were taken using a 10× objective and representative higher power magnified images with a 30× objective using an Olympus IX81 fluorescence microscope (Olympus America Inc., Center Valley, PA). The montage images were overlapped and assembled using Adobe PhotoShop software (Adobe Systems Inc., San Jose, CA).

### Western blot analysis

Corneal tissues were minced on ice and lysed in ice-cold lysis buffer containing 5 mM NaF, 1 mM PMSF, 1 mM DTT, 20 mM HEPES, 1 mM EDTA, 400 mM NaCl, 1 mM EGTA, 0.1% NP-40, and a protease inhibitor cocktail (Roche, Indianapolis, IN). The lysate was flash-frozen and thawed 3 times, vortexed, and finally centrifuged for 10 min at 10,000× g to sediment debris. The soluble fraction of retinal tissue samples was extracted using an ice-cold lysis buffer containing Tris-buffered saline (TBS), 1% NP-40, 200 mM NaCl with 5 mM NaF, 1 mM PMSF, 1 mM DTT, and a protease inhibitor cocktail. Equal amount of proteins were subjected to SDS–PAGE on 10% polyacrylamide gels (Biorad, Hercules, CA) and transferred to a PVDF membrane. Blots were probed with anti-rabbit GFAP antibody (1: 20,000; Abcam) overnight at 4 °C. After extensive washing and incubation with respective secondary antibody conjugated to horseradish peroxidase, the membrane was developed using chemiluminescent reagents (Amersham, Piscataway, NJ) and exposed to X-ray film.

### Filament analysis

GFAP filaments were analyzed as previously reported [21]. Briefly, digital images of GFAP stained retinas from thin tissue sections were converted to black and white images. The lengths of filaments in Müller glia were measured using in-house software that we have developed and statistical analysis was performed using the unpaired Student *t* test [21]. Filament density was calculated by measuring the total numbers of filament structures in each treatment group divided by the number of slides (n=15 per group).

## Results

### Simultaneous induction of corneal neovascularization and retinal gliosis after eye alkali burn injury

**Figure 1:**
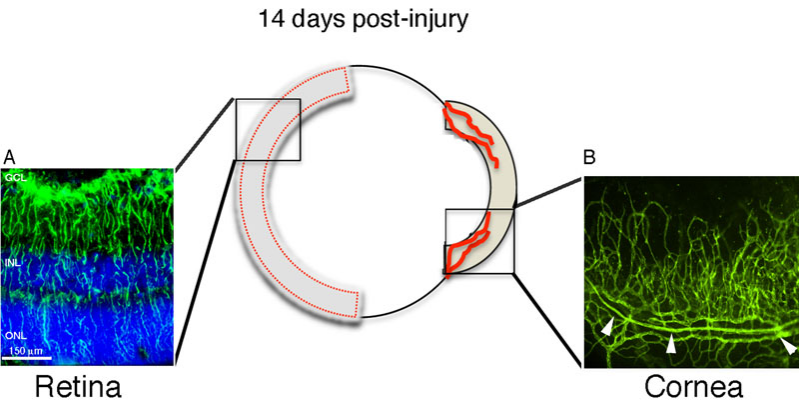
Mouse alkali burn injury model for corneal neovascularization and retinal gliosis. Corneal alkali injury with limbal and corneal epithelial cell debridement induces two distinct ocular pathologies that are robustly elaborated at 14 days post injury: 1) gliosis in the retina (left panel) and 2) neovascularization in the cornea (right panel). **A**: Reactive Müller cells labeled with glial fibrillari acidic protein (GFAP) antibody (green) in injured sample from thin tissue section. Cell nuclei were stained with 4′, 6-diamidino-2-phenylindole (DAPI, blue). **B**: Representative image of a segment from whole-mount injured cornea labeled with CD-31^+^ antibody (green) showing neovascularization from pre-existing limbal vessels (arrowheads). The cornea image was taken at 10× magnification and the retina image at 30×. Abbreviations: GCL represents glial cell layer; INL represents inner nuclear layer; ONL represents outer nuclear layer; Epi represents epithelium.

We previously reported the induction of corneal angiogenesis [20] and retinal gliosis [21] after alkali injury in mice from a 129/Svev background. Since mouse strains can exhibit different responses to corneal angiogenic stimuli [18], we also validated this model in C57BL/6J mice, a common laboratory strain frequently employed in drug testing. In addition, we have extended the time course to 14 days post-alkali injury to assess the temporal dynamics of retinal gliosis in this model, since the peak of corneal inflammatory angiogenesis occurs at 14 days after injury [24]. Two distinct pathologies of retinal gliosis (Figure 1A) and corneal neovascularization (Figure 1B) were robustly sustained at 14 days post-injury in the injured mice. Mice did not exhibit any adverse behavioral effects and were indistinguishable from uninjured controls after day 3 post-injury. The corneal epithelium healed without drug intervention, which is important for investigations designed to test new drug leads, as this eliminates potential drug-drug interactions that could alter the effectiveness of the treatment modalities.

### Assessment of corneal neovascularization 14 days post-injury

**Figure 2:**
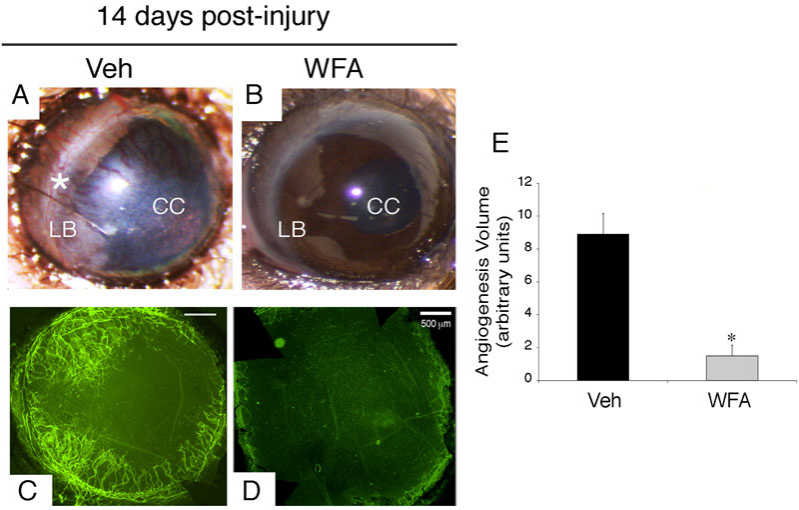
Neovascularization in the cornea at 14 days post-injury. Mice were subjected to corneal alkali injury with limbal and corneal epithelial cell debridement and treated daily with dimethyl sulfoxide (DMSO, Veh) or 2 mg/kg withaferin A (WFA) for 14 days. **A-B**: Representative images of anterior segment showing phenotypic representation of corneal neovascularization with and without drug treatment. An extensive network of new blood vessels extends from the limbus (LB, asterisk) into the central cornea (CC) in Veh sample. **C-D**: Whole mount staining of corneas labeled with anti-CD31-fluorescent antibody (green) show an extensive network of new blood vessels in vehicle (Veh) sample with potent inhibition in sample treated with WFA (**D**). **E**: Quantification of neovascularization from each group of mice (n=14) was performed as previously described [20]. Error bars represent standard deviation (SD); * p<0.05 by the Mann–Whitney U test.

As expected, at 14 days post-injury, the corneas of injured mice reveal robust growth of new blood vessels that extended from the limbus (LB) to the central cornea (CC), as shown in representative images (Figure 2A). Staining of whole-mount corneas with anti-CD31-fluorescent antibody clearly confirmed the presence of new blood vessels in the injured tissue (Figure 2C). The delivery of the potent angiogenesis inhibitor WFA [22] inhibited neovascularization when compared with the vehicle treated group at day 14 post-injury (Figure 2C,D). Quantification of neovascularization from each group of mice (n=14) confirmed the microscopic observations (Figure 2E).

### Assessment of retinal gliosis 14 days post-injury

**Figure 3:**
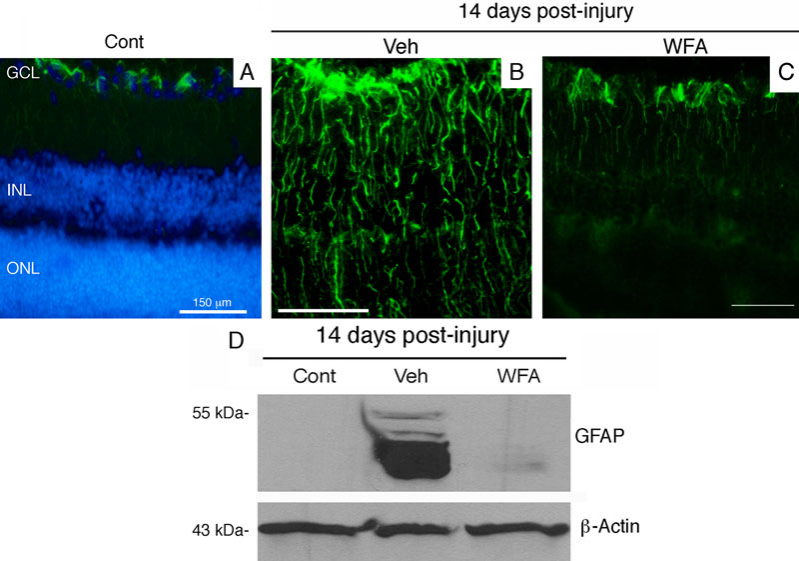
Fourteen days post-injury gliosis in the retina. Mice were subjected to corneal alkali injury with limbal and corneal epithelial cell debridement and treated daily with dimethyl sulfoxide (DMSO, Veh) or 2 mg/kg withaferin A (WFA) for 14 days. **A-C**: Tissue sections of eyes from uninjured (Cont), injured (veh) and WFA treated mice were stained with antibodies to glial fibrillary acidic protein (GFAP; green). Nuclei were stained with 4′, 6-diamidino-2-phenylindole (DAPI, blue) and fluorescent staining was visualized with a Nikon TE2000 microscope. **D**: western blot analysis of GFAP expression in soluble extracts of retina samples from uninjured (cont), injured (Veh) and WFA treated mice. Abbreviations: GCL represents ganglion cell layer; INL represents inner nuclear layer; ONL represents outer nuclear layer.

**Figure 4:**
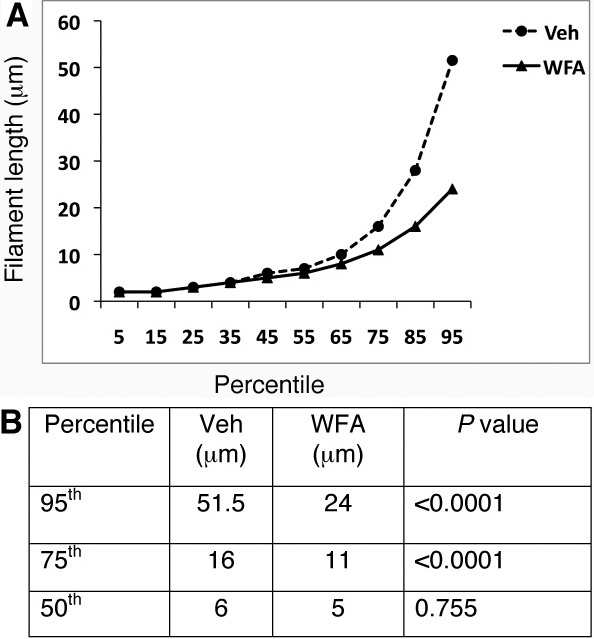
Quantitative assessment of glial fibrillary acidic protein (GFAP) filaments. Mice were subjected to corneal alkali injury with limbal and corneal epithelial cell debridement and treated daily with dimethyl sulfoxide (DMSO, Veh) or 2 mg/kg withaferin A (WFA) for 14 days. Tissue sections of injured eyes from vehicle-treated and WFA-treated mice (n=15/group) were stained with antibodies to GFAP and filaments quantified as previously reported [21]. Graphical representations of GFAP filament lengths as percentiles are shown between vehicle-treated and WFA-treated samples (**A**). Significant differences were noted above the 50th percentiles between these two groups (**B**).

**Figure 5:**
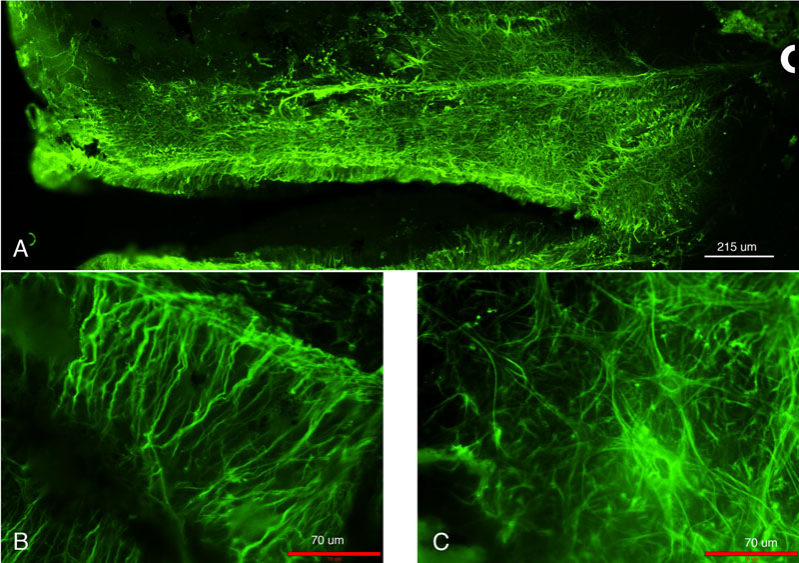
Glial fibrillary acidic protein (GFAP) localization in retinal flat-mounts of alkali injured mice at 14 days post-injury. **A**: Prominent GFAP expression as observed in this image montage is visible throughout the retina most notably upregulated in astrocytes. **B**: Enlarged image of activated Muller cells. **C**: Representative enlarged image of astrocytes showing GFAP staining. For orientation purposes, the optic nerve is marked with a white semi-circular line. The scale bar in the montage image (**A**) is 215 μm. The scale bars in the enlarged images are 70 μm.

Retinas of mice injured by alkali displayed abundant expression of polymerized GFAP filaments in Müller glial cells at day 7, as revealed in tissue sections stained for this gliosis marker [21]. This expression was also associated with a corresponding increase in soluble GFAP expression in the retina, associated with Müller cells entering into the cell cycle [21]. GFAP expression as polymerized filaments was robustly present in the Müller glial cells (Figure 3B). An increase in expression of GFAP as its soluble form was also maintained (Figure 3D). The GFAP expression as polymerized IFs stained the entire retina and was maintained at this high expression level from day 7 post-injury [21]. When mice were treated with WFA, soluble GFAP isoforms/variants were found to be potently downregulated (Figure 3D), which led to effects on GFAP polymerization, causing IFs to show a fragmented phenotype (Figure 3C). We assessed the length of all filamentous structures stained for GFAP and interrogated the growth profiles as percentiles [21]. We found that WFA treated mice overall had shorter GFAP-stained filaments, with lengths only approximately half those of vehicle treated mice (Figure 4). The WFA-treated mice had a 5.7 fold reduction in GFAP-staining filament densities compared to vehicle-treated mice (p<0.001). This was in keeping with our observation that the longer period of drug treatment (14 days) almost completely abrogated the expression of soluble GFAP (Figure 3D) whereas only a partial reduction occurred at 7 days post-injury [21]. This targeting of soluble GFAP by WFA [21] led to a reduction in filamentous forms of GFAP at 14 days post injury. GFAP induction in response to injury was also demonstrated by flatmount staining, which showed extensive upregulation in the central and peripheral retina (Figure 5A). Higher power magnified images (Figure 5B,C) revealed upregulation of GFAP in astrocytes and Müller glia, where the latter could be discerned by their parallel radial processes observable in those regions that were not fully flattened by tissue mounting. In summary, alkali burn injury robustly induced retinal gliosis in mouse retinas and this phenotype was manifested even at 14 days post-injury. WFA treatment potently downregulated soluble GFAP and also qualitatively and quantitatively attenuated polymerized GFAP IF expression in the mouse retina.

## Discussion

We have developed an ocular chemical injury model that simultaneously elicits anterior and posterior segment pathology in the mouse eye. Corneal neovascularization and retinal gliosis develop by day 7 [20,21] and remain robustly present at 14 days post-injury. This model reduces the number of mice required, it is quick, and it is well suited for testing the inhibitory effects of drugs on angiogenesis and gliosis.

The first step of the procedure (alkali burn injury, followed by mechanical scraping of the corneal epithelium) requires only a moderate level of expertise for ocular researchers, and with practice, the technique ensures very high success rates. Different time course experiments can be planned and the progression of the disease (corneal neovascularization) can be monitored daily, even without anesthesia, by direct visual inspection of the eye under a dissecting microscope (Figure 2A-C) by an investigator skilled in mouse handling procedures. In the second step of the procedure (immunohistochemistry analysis of neovascularization and gliosis), upon enucleation of the eye, the corneal and retinal tissues can be dissected out and analyzed separately for western blot analysis [21] or whole mount staining [20,25]. Alternatively, the whole eye can be embedded and cryosectioned for immunostaining with single or multiple antibodies [21]. The immunohistochemical labeling of samples for gliosis markers (GFAP) and neovascularization (CD31) is robust because these proteins are highly expressed in Müller glia and blood vessels, respectively. The staining of these tissues remains for long periods (over a week when samples are stored at 4 °C in cold PBS), which enables sequentially analysis of multiple batches with little or no change in staining results. The third step of the procedure (software analysis) allows quantification of the stress response of the Müller glia by image analysis. For this, we typically collect 15–20 representative images at the same magnification per test sample, obtained from several different animals. In this injury model, GFAP staining is present in virtually all Müller glia across the entire retina, which is also corroborated by flatmount staining. Just as CD31 staining of corneal whole mounts is represented over the entire corneal tissue, global expression of GFAP expression in Müller glia makes this model very useful for quantitative analysis of gliosis.

The concept of divide and rule has enabled the resolution of many challenging biologic problems. It is an enabling strategy for complex human diseases, where targeting the most vulnerable and sometime distal pathological process of a complex disease can afford significant therapeutic control. For instance, the targeting of angiogenic growth factors to control tumor proliferation, or the blocking of choroidal neovascularization to slow wet-AMD, show how mechanistic insight into the vulnerability of the proliferating blood vessels that support tumor growth [26] or neurodegenerative eye diseases [27] can give rise to clinical treatments for the management of these diseases. In the case of AMD, the success of antiangiogenic drugs confirms that this vasculature is responsive to therapeutics and that control of vision loss can be achieved. In contrast, blocking the insidious glial fibrosis of the eye remains a challenge, not only for AMD [14,28], but for numerous other retinal blinding conditions as well [29].

For this reason, combination treatments that simultaneously block vascular and glial proliferation in neurodegenerative diseases like AMD might be required, thereby necessitating synergistic therapies [3]. These types of investigations are quite expensive because the drug-drug interactions of these drug combinations have to be taken into account. The mouse alkali injury model described here can provide a quick in vivo method for screening combinations of drugs, or to test polypharmacological activities of compounds (e.g., WFA) on glial and blood vessel target(s). Finally, because angiogenesis and gliosis are elicited in this model in response to endogenous mediators, we believe this affords a natural setting where the use of genetics and pharmacology can be combined for the study of the mechanisms of drugs and for validation of their targets [30].
